# Supplementary material for: P-TRAP: a Panicle Trait Phenotyping tool
Source: BMC Plant Biol. 2013 Aug 29;13:122. doi: 10.1186/1471-2229-13-122 (PMC3848748; doi:10.1186/1471-2229-13-122)
Supplement: Additional file 7 — P-TRAP source code. The Netbeans project that contains the source code of the application. [file 1471-2229-13-122-S7.zip › javadoc.zip!/javadoc/org-bioalg-imageprocessor/org/bioalg/imageprocessor/ImageIO.html]

ImageIO (Image Processor)


JavaScript is disabled on your browser.


- Package
- Class
- Tree
- Deprecated
- Index
- Help

*org.bioalg.imageprocessor 1.0*

- Prev Class
- Next Class

- Frames
- No Frames

- All Classes

- Summary:
- Nested |
- Field |
- Constr |
- Method

- Detail:
- Field |
- Constr |
- Method


org.bioalg.imageprocessor

## Class ImageIO

- java.lang.Object
- - org.bioalg.imageprocessor.ImageIO

- ---

    

  ```
  public class ImageIO
  extends java.lang.Object
  ```

  The Input Output operations of the image based on both generic java imaging
  and JAI imageing. Please make sure you have included the jar file of the JAI
  before you can compile this file.

  Author:
  :   aanjos and Faroq Al-tam

- - ### Constructor Summary

    Constructors

    Constructor and Description

    `ImageIO()`
  - ### Method Summary

    Methods

    | Modifier and Type | Method and Description |
    | --- | --- |
    | `static boolean` | `convertType(java.io.File sourceFile, java.io.File destinationFile, java.lang.String imageType)` Convert image types |
    | `static javax.media.jai.TiledImage` | `createImage(int[][] intMatrix, int numberOfBands, int dataType)` Create an image given the data in an integer matrix. |
    | `static javax.media.jai.TiledImage` | `createImageDouble(double[][] intMatrix, int numberOfBands, int dataType)` Create a double-valued image. |
    | `static AImage` | `getImageAsAImage(javax.media.jai.PlanarImage pImage)` Convert a `PlanarImage` to a `AImage`. |
    | `static AImage` | `getImageAsGrayscaledAImage(java.io.File file)` Load and return a grayscale version of the image |
    | `static ColoredImage` | `LoadColoredImage(java.io.File file)` Load a colored image form a file. |
    | `static void` | `saveAImageAsImage(AImage matrix, java.lang.String filename)` Store a `AImage` to a file. |
    | `static void` | `saveAImageAsImage(AImage matrix, java.lang.String filename, java.lang.String type)` Store the input image to a file with a given type. |
    | `static void` | `saveAImageAsImageFloat(AImage matrix, java.lang.String filename)` Store the input image as floating point. |

    - ### Methods inherited from class java.lang.Object

      `clone, equals, finalize, getClass, hashCode, notify, notifyAll, toString, wait, wait, wait`

- - ### Constructor Detail


    - #### ImageIO

      ```
      public ImageIO()
      ```
  - ### Method Detail


    - #### getImageAsGrayscaledAImage

      ```
      public static AImage getImageAsGrayscaledAImage(java.io.File file)
      ```

      Load and return a grayscale version of the image

      Parameters:
      :   `file` - the image file

      Returns:
      :   an `AImage` object.


    - #### LoadColoredImage

      ```
      public static ColoredImage LoadColoredImage(java.io.File file)
      ```

      Load a colored image form a file.

      Parameters:
      :   `file` - the input `File`

      Returns:
      :   a `ColoredImage` object.


    - #### getImageAsAImage

      ```
      public static AImage getImageAsAImage(javax.media.jai.PlanarImage pImage)
      ```

      Convert a `PlanarImage` to a `AImage`.

      Parameters:
      :   `pImage` - the planarimage

      Returns:
      :   a `AImage`.


    - #### saveAImageAsImage

      ```
      public static void saveAImageAsImage(AImage matrix,
                           java.lang.String filename)
      ```

      Store a `AImage` to a file.

      Parameters:
      :   `matrix` - the input image.
      :   `filename` - the file where to save the image.


    - #### saveAImageAsImage

      ```
      public static void saveAImageAsImage(AImage matrix,
                           java.lang.String filename,
                           java.lang.String type)
      ```

      Store the input image to a file with a given type.

      Parameters:
      :   `matrix` - the input image
      :   `filename` - the file where the image has to be stored
      :   `type` - the file type.


    - #### saveAImageAsImageFloat

      ```
      public static void saveAImageAsImageFloat(AImage matrix,
                                java.lang.String filename)
      ```

      Store the input image as floating point.

      Parameters:
      :   `matrix` - input image
      :   `filename` - the file where to save the image.


    - #### createImage

      ```
      public static javax.media.jai.TiledImage createImage(int[][] intMatrix,
                                           int numberOfBands,
                                           int dataType)
      ```

      Create an image given the data in an integer matrix.

      Parameters:
      :   `intMatrix` - the data
      :   `numberOfBands` - the number of bands
      :   `dataType` - the datatype of the pixels

      Returns:


    - #### createImageDouble

      ```
      public static javax.media.jai.TiledImage createImageDouble(double[][] intMatrix,
                                                 int numberOfBands,
                                                 int dataType)
      ```

      Create a double-valued image.

      Parameters:
      :   `intMatrix` - the data grid.
      :   `numberOfBands` - the number of bands
      :   `dataType` - the type of the data.

      Returns:
      :   a `TiledImage` object


    - #### convertType

      ```
      public static boolean convertType(java.io.File sourceFile,
                        java.io.File destinationFile,
                        java.lang.String imageType)
      ```

      Convert image types

      Parameters:
      :   `sourceFile` - the input file
      :   `destinationFile` - the output file
      :   `imageType` - the type to save with

      Returns:
      :   true if success, false otherwise.


- Package
- Class
- Tree
- Deprecated
- Index
- Help

*org.bioalg.imageprocessor 1.0*

- Prev Class
- Next Class

- Frames
- No Frames

- All Classes

- Summary:
- Nested |
- Field |
- Constr |
- Method

- Detail:
- Field |
- Constr |
- Method
